# Rapid species identification of pathogenic bacteria from a minute quantity exploiting three-dimensional quantitative phase imaging and artificial neural network

**DOI:** 10.1038/s41377-022-00881-x

**Published:** 2022-06-23

**Authors:** Geon Kim, Daewoong Ahn, Minhee Kang, Jinho Park, DongHun Ryu, YoungJu Jo, Jinyeop Song, Jea Sung Ryu, Gunho Choi, Hyun Jung Chung, Kyuseok Kim, Doo Ryeon Chung, In Young Yoo, Hee Jae Huh, Hyun-seok Min, Nam Yong Lee, YongKeun Park

**Affiliations:** 1 Department of Physics, Korea Advanced Institute of Science and Technology, Daejeon, 34141 Republic of Korea; 2 KAIST Institute for Health Science and Technology, KAIST, Daejeon, 34141 Republic of Korea; 3 Tomocube Inc., Daejeon, 34109 Republic of Korea; 4 Smart Healthcare & Device Research Center, Samsung Medical Center, Sungkyunkwan University School of Medicine, Seoul, 06351 Republic of Korea; 5 Graduate School of Nanoscience and Technology, Korea Advanced Institute of Science and Technology, Daejeon, 34141 Republic of Korea; 6 Department of Biological Sciences, Korea Advanced Institute of Science and Technology, Daejeon, 34141 Republic of Korea; 7 Department of Emergency Medicine, Bundang CHA Hospital, Seongnam-si, Gyeonggi-Do 13496 Korea; 8 Division of Infectious Diseases, Department of Internal Medicine, Samsung Medical Center, Sungkyunkwan University School of Medicine, Seoul, 06351 Republic of Korea; 9 Department of Laboratory Medicine, Seoul St. Mary’s Hospital, College of Medicine, The Catholic University of Korea, Seoul, 06591 Republic of Korea; 10 Department of Laboratory Medicine and Genetics, Samsung Medical Center, Sungkyunkwan University School of Medicine, Seoul, 06351 Republic of Korea; 11 Present Address: Department of Applied Physics, Stanford University, Stanford, CA 94305 USA; 12 Present Address: Department of Physics, Massachusetts Institute of Technology, Cambridge, MA 02139 USA

**Keywords:** Optical sensors, Microscopy, Biophotonics

## Abstract

The healthcare industry is in dire need of rapid microbial identification techniques for treating microbial infections. Microbial infections are a major healthcare issue worldwide, as these widespread diseases often develop into deadly symptoms. While studies have shown that an early appropriate antibiotic treatment significantly reduces the mortality of an infection, this effective treatment is difficult to practice. The main obstacle to early appropriate antibiotic treatments is the long turnaround time of the routine microbial identification, which includes time-consuming sample growth. Here, we propose a microscopy-based framework that identifies the pathogen from single to few cells. Our framework obtains and exploits the morphology of the limited sample by incorporating three-dimensional quantitative phase imaging and an artificial neural network. We demonstrate the identification of 19 bacterial species that cause bloodstream infections, achieving an accuracy of 82.5% from an individual bacterial cell or cluster. This performance, comparable to that of the gold standard mass spectroscopy under a sufficient amount of sample, underpins the effectiveness of our framework in clinical applications. Furthermore, our accuracy increases with multiple measurements, reaching 99.9% with seven different measurements of cells or clusters. We believe that our framework can serve as a beneficial advisory tool for clinicians during the initial treatment of infections.

## Abstract (web summary)

Label-free rapid deep-learning-based identification of bacterial species that classifies 3D refractive index tomograms into the species.

## Graphical abstract

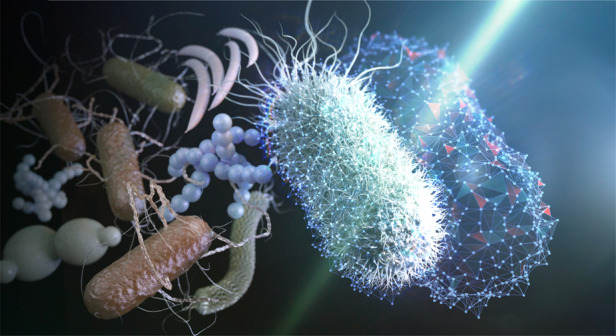

## Introduction

Infections by microorganisms are a global healthcare issue that is associated with a large number of deaths and a significant amount of expenses. Notably, bacteria account for approximately half of the reported cases of infections^1^, as well as a large portion of the entire healthcare spending^2^. Hence, effectively treating this widespread and possibly deadly illness has been a long-sought goal in the clinical society.

Multiple studies indicate that an antibiotic treatment appropriate to the pathogen, during the early hours of an infection, can significantly reduce the mortality^3,4^. In clinical settings, however, early antibiotic treatments are commonly empirical and imperfect, mainly due to the long turnaround time of routine microbial identification^5,6^, resulting in increased mortality risk^7^.

The typical turnaround time of the routine microbial identification is over 24 h^8^. Conventional approaches including culture tests are often nonspecific as well as time-consuming, despite being relatively simple to perform^9^. Molecular diagnostic methods screen for genetic materials in a shorter duration, yet they are not scalable for arbitrary pathogens^8^. In recent days, matrix-assisted laser desorption/ionization time-of-flight mass spectroscopy (MALDI-TOF MS) serve as the gold standard of microbial identification. MALDI-TOF MS detects the molecular markers of bacteria^8,9^ but only when the sample quantity is detectable, which is commonly satisfied after 24 h of culture.

Image-based methods have also been implemented to promptly detect or identify bacteria from a low quantity. Fluorescence microscopy has often been utilized in detecting and counting individual bacteria^10^. More recently, fluorescence in situ hybridization has allowed screening for certain types of bacteria, by specifically labeling genomic patterns^11,12^. However, fluorescence imaging entails destructive chemical alteration of the sample, as well as requiring optimally manufactured probes for high specificity. Label-free alternatives including autofluorescence microscopy have been adopted for bacterial detection to circumvent the drawbacks of labeling^13,14^, but at a specificity restricted to the variation in the intrinsic fluorophores.

In this study, we tackle the challenge of rapid microbial identification by exploiting three-dimensional (3D) quantitative phase imaging (QPI) and image classification based on an artificial neural network (ANN). 3D QPI is a label-free imaging technique that measures the 3D refractive index (RI) tomogram of a live cell and has been actively employed in quantitative cell profiling^15–19^.

Our unprecedented utilization of 3D QPI and ANN for bacterial identification achieves 82.5% accuracy in determining the species from a single bacterial cell or cluster. The accuracy increases with 3D QPI measurements of multiple specimens, reaching 99.9% with seven different measurements. We note that this accuracy is obtained between 19 major species of bacteria that account for bloodstream infections (BSIs)^20–22^, further underlining the potential in clinical applications. This exceptional performance from a minute quantity of bacteria suggests that the proposed method can guide the early antibiotic treatment prior to the time-consuming culture process.

## Results

**Fig. 1:**
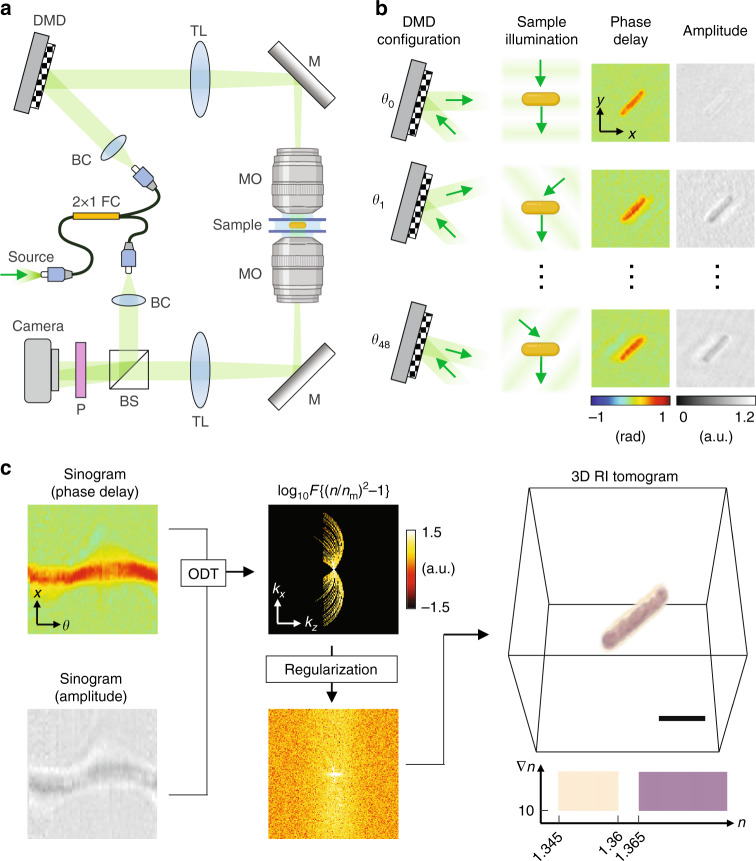
Three-dimensional (3D) QPI measurement of bacteria. **a** The optical system is based on a simplified Mach-Zehnder interferometer equipped with a DMD. BC: beam collimator. BS: beam splitter. CL: condenser lens. FC: fiber coupler. LP: linear polarizer. MO: microscope objective lens. TL: tube lens. **b** Holograms including both the phase delay and the amplitude are measured while altering the illumination angle using the DMD. **c** The 3D RI tomogram is acquired by integrating the sinogram into the scattering potential via optical diffraction tomography, followed by an iterative regularization

The workflow of the 3D QPI in the identification framework is illustrated in Fig. 1. Our 3D QPI system, which is commercialized and dubbed holotomography (HT-2H, Tomocube Inc., Daejeon, Republic of Korea), utilizes Mach-Zehnder laser interferometry equipped with a digital micromirror device (DMD) as shown in Fig. 1a. The DMD scans the illumination angle and the 3D refractive index (RI) tomogram is reconstructed from the sinogram of 2D QPI measurements under the principle of optical diffraction tomography (Fig. 1b, c)^23^.

**Fig. 2:**
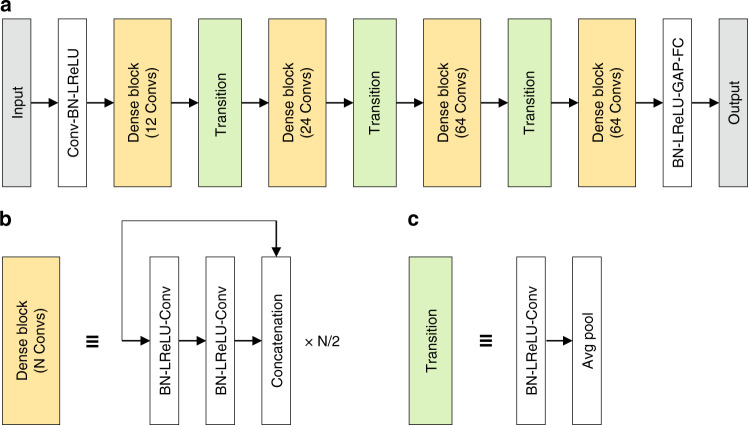
The structure of the ANN utilized in our framework. **a** Four dense blocks and transition units between adjacent dense blocks represent the overall structure. Other elements include the initial 3D convolution operation (Conv) of 3 × 3 × 3 kernels and a stride of 2 × 2 × 2, batch normalization (BN), leaky rectified linear units (LReLU), global average pooling (GAP), and fully connected operation (FC). **b** A dense block repeats a pair of Convs followed by a concatenation of the feature map. In each pair of Convs the first one is of 1 × 1 × 1 kernels and the second one is of 3 × 3 × 3 kernels, while the stride is 1 × 1 × 1 for both. **c** The transition units shift the scale of the feature extracted by convolution. The Conv in each transition unit is of 1 × 1 × 1 kernels and a 1 × 1 × 1 stride

The 3D RI tomogram is then classified into one of the 19 species, through a trained ANN. The training process involves gradient-based optimization of the network parameters, using the training dataset whose species are known. Our implementation of ANN mainly consists of 3D convolution operations for effective recognition of the 3D structure in 3D RI tomograms (Fig. 2). More specifically, the dense connections between the convolution operations induce the ANN to revisit the feature maps of the shallower layers even at the deep layers^24^.

The key function of this identification framework is to identify the species of the bacteria from single to few cells. It can provide preliminary results during the early stages of infections before the diagnostic evidence from gold standard methods is available dozens of hours later. Incorporation of the proposed framework into the gold standard routine is practicable since it operates without destroying nor chemically modifying the bacteria.

### 3D QPI measurement of bacteria

**Fig. 3:**
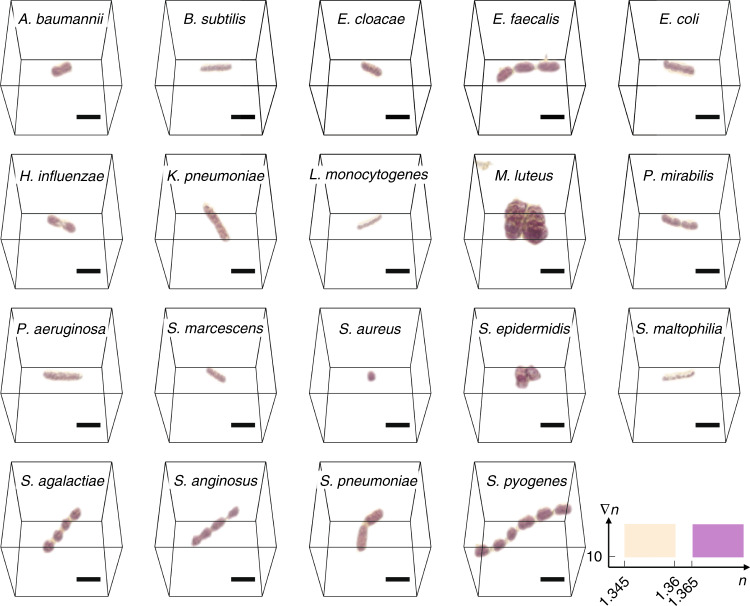
Three-dimensional (3D) RI tomograms of bacterial BSI pathogens. Representative tomograms addressed in our study are rendered in 3D. Each tomogram represents an individual species of bacterial BSI pathogens. Scale bar = 2 μm

A database of 3D RI tomograms was established from the isolates of 19 BSI-related bacterial species (Fig. 3). The database comprised a total of 10,556 3D RI tomograms, where each tomogram contained a single bacterium or several adhering bacteria. 3D QPI effectively conveyed the 3D structure of the bacteria, and some characteristic morphologies were visible in the 3D RI tomograms, e.g., cellular chains of streptococci. The species and the corresponding numbers of tomograms are as follows: *Acinetobacter baumannii* (664), *Bacillus subtilis* (515), *Enterobacter cloacae* (541), *Enterococcus faecalis* (526), *Escherichia coli* (600), *Haemophilus influenzae* (511), *Klebsiella pneumoniae* (525), *Listeria monocytogenes* (632), *Micrococcus luteus* (247), *Proteus mirabilis* (517), *Pseudomonas aeruginosa* (596), *Serratia marcescens* (519), *Staphylococcus aureus* (558), *Staphylococcus epidermidis* (559), *Stenotrophomonas maltophilia* (549), *Streptococcus agalactiae* (537), *Streptococcus anginosus* (644), *Streptococcus pneumoniae* (566), and *Streptococcus pyogenes* (750). The majority tomograms of bacilli, i.e., rod-shaped bacteria, contained single bacterial cells. On the other hand, most of cocci and coccobacilli, i.e., spherical and ovoid bacteria, respectively, were in the form of clusters of several adhering bacteria. For instance, the specimens belonging to the genus *Streptococcus* are mostly found as chains of multiple adhering bacteria; a feature that the genus is characterized with. 3D QPI also facilitates the calculation of biophysical properties of each specimen (see section 1 of Supplementary Information), owing to the quantitative contrast related to the sample composition.

### Identification of pathogens using a single tomogram

**Fig. 4:**
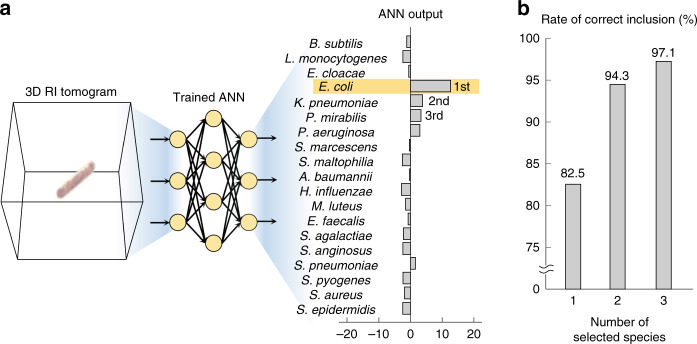
Species identification using a single 3D RI tomogram. **a** The ANN processes a given 3D RI tomogram and results in the output indicating the 19 species. **b** Pinpointing the single most likely species based on the ANN output is 82.5% accurate in the blind test. The risk of omitting the ground true species is reduced by considering multiple likely species indicated in the ANN output

With a single 3D RI tomogram, the proposed framework achieved a blind test accuracy of 82.5% in species identification. This single-measurement accuracy is comparable to the rate of correct species identification obtained using MALDI-TOF MS with a sufficient number of bacteria^25^. The high performance was realized despite the limited amount of samples, by statistically utilizing the detailed 3D morphologies of the bacteria. Namely, each neuron in the ANN was distinctly activated based on the morphology of the input tomogram, as the result of the training process. This led the ANN output to be related to the conditional probability of the species given the input tomogram and the training data distribution (Fig. 4a).

We note that this accurate single-measurement identification is the product of both 3D QPI and ANN, which rigorously measure and recognize the morphologies, respectively. To verify this, variant frameworks were implemented by altering the imaging strategy and the algorithm (see sections 2–4 of Supplementary Information). The performance of species identification dramatically decreased when 3D QPI was replaced with 2D QPI or 2D QPI sinogram, as well as when the ANN was replaced with a conventional machine learning algorithm^26^.

The omission of the correct species could be further prevented at the expense of specificity. Namely, the correct species can be indicated at a higher rate by taking more than one species as the possible pathogen; we refer to this rate that the correct species is included in the *N* most likely species as the top-*N* accuracy. The top-2 accuracy and top-3 accuracy of the proposed framework were 94.3% and 97.1%, respectively (Fig. 4b). In clinic, although this trade-off itself is not unexpected, lowering risk with such strategies would be favorably considered whereas the loss of specificity can be buffered based on other indications, including characteristic symptoms and environmental evidence. Also, the sharp mitigation of the omission rate also underlines that the ANN robustly extracted features related to the correct species, even in the misidentified data. This robust feature extraction ability was also indicated by comparing the contrast of ANN outputs for the correctly and incorrectly identified data (see section 5 of Supplementary Information).

### Error in identification using a single tomogram

**Fig. 5:**
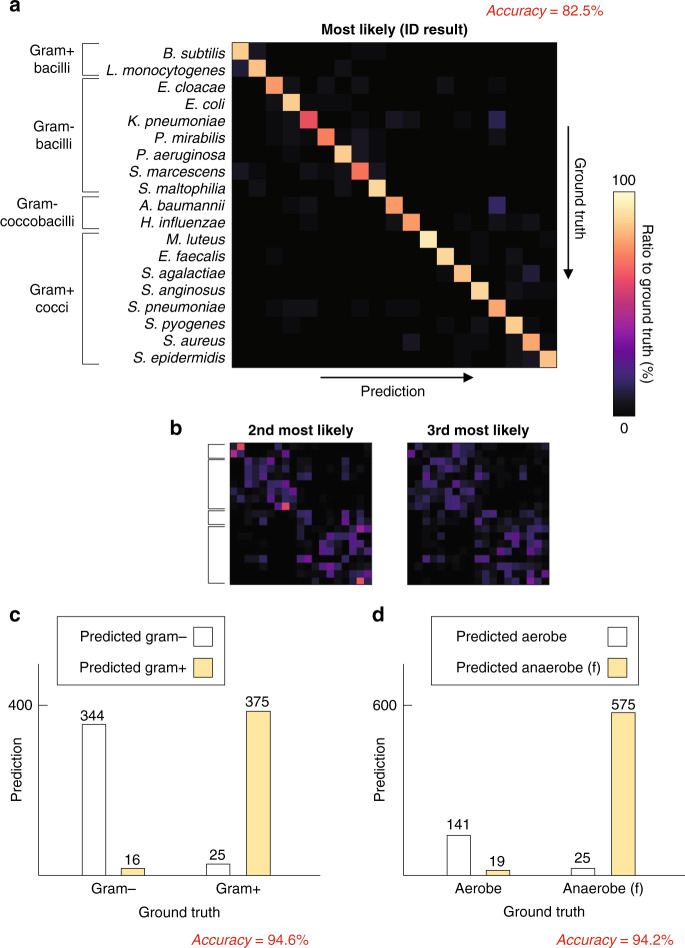
Distribution of error in the species identification using a single 3D RI tomogram. **a** The confusion matrix visualizes the overall performance and the frequent errors in the blind test dataset. The row and column indices correspond to the ground truth and the prediction, respectively. The indices of the 19 species are ordered to reflect the common bacterial categories. **b** The distribution of the second and the third most likely species further visualizes the interspecific similarity recognized by the trained ANN. **c**, **d** Individual tomograms are categorized under broader groups including gram-stainability and respiratory metabolism using a modified ANN for each task

To characterize the distribution of errors, the blind test result for the entire test dataset was investigated using the confusion matrix (Fig. 5a). The most frequent errors included the misidentification of *A. baumannii* as *S. pneumoniae*, *K. pneumoniae* as *S. pneumoniae*, *S. agalactiae* as *S. aureus*, and *L. monocytogenes* as *B. subtilis*. Notably, the misidentification of thick bacilli and coccobacilli as *S. pneumoniae* contributed to a large portion of the error. This is in consistency with the relatively elongated morphology of *Streptococcus pneumoniae* compared to other cocci^27,28^. The overall identification performance varied among different species of bacteria. Among the 19 species, *M. luteus* was identified with both the highest sensitivity (95.0%) and specificity (100%). *K. pneumoniae* was the least sensitively identified species (62.5%), whereas *S. peumoniae* was the least specifically identified species (97.8%). The distribution of sensitivity and specificity in identifying each species are presented in more detail in section 6 of Supplementary Information.

The distribution of the second and third most likely species provided further insights regarding interspecific similarities (Fig. 5b). These plots visualize how similar the test data of different species are, concerning the features extracted by the ANN. Notably, a group of multiple species with morphological resemblance can be outlined as a cluster. The species of bacilli form a large cluster while the rest of the 19 species form another large cluster. In addition, *E. cloacae*, *E. coli*, and *K. pneumoniae*, namely, the species belonging to the family *Enterobacteriaceae*, showed a distinct clustering amidst other species of bacilli.

Apart from species identification, the proposed framework accurately performed common categorizations of bacteria from a single 3D QPI measurement. Accuracies of 94.6% and 94.2% were achieved in distinguishing between Gram-negative and positive bacteria, and between aerobic and facultatively anaerobic bacteria, respectively (Fig. 5c, d). This suggests the capability to distinguish bacteria in different standards, after training the ANN accordingly while maintaining the workflow.

### Identification of pathogens using multiple tomograms

**Fig. 6:**
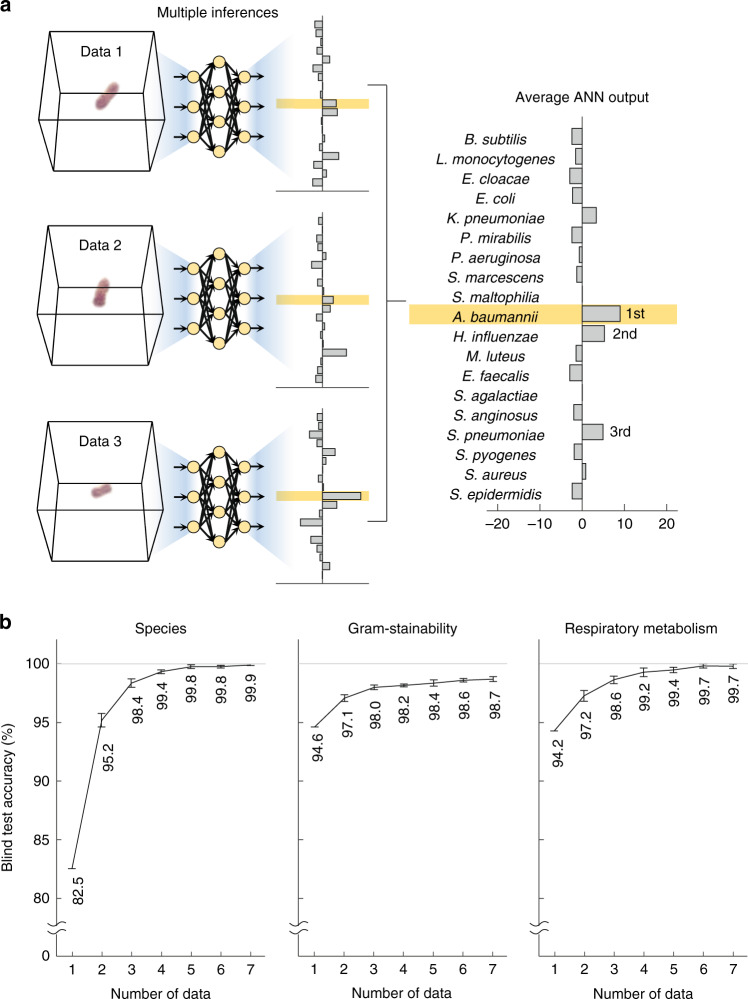
Species identification based on multiple measurements of 3D RI tomograms. **a** Securing a higher accuracy by taking the average of ANN outputs resulting from multiple tomograms. The highlighted species indicate the correct species in each ANN output. **b** Reduction of error in classifying the species, gram-stainability, and respiratory metabolism. The error reduction is sharper than a simple reciprocal function owing to the feature-extracting ability of the artificial neural network

While the single-measurement performance of the proposed framework was comparable to that of the gold standard methods, securing more samples further increases the identification accuracy. The identification based on multiple measurements of 3D RI tomograms was realized by taking the average of the ANN outputs resulting from each of the individual 3D RI tomograms (Fig. 6a). The accuracy of species identification rose from 84.5% to 95.2%, 98.4%, and 99.9%, when reflecting two, three, and seven tomograms, respectively (left column, Fig. 6b). The error rate dropped more sharply than a simple reciprocal function of the sample quantity. This dramatic gain in the accuracy was attributable to the robust feature-extracting ability of the ANN. The correct species were strongly indicated in the ANN output even in the misidentified cases, as underlined in the abovementioned trade-off between the sensitivity and specificity; this can be seen from example data and outputs displayed in Fig. 6a where the multi-measurement identification is accurate even when the majority of the individual tomograms are misclassified.

The multi-measurement strategy was also applied to the categorization between Gram-positive and negative bacteria, and between aerobic and facultatively anaerobic bacteria (center and right columns, Fig. 6b). Although a larger sample quantity led to higher performances in these categorizations as well, the gain in accuracy was not as significant as in the species identification. The two standards for categorization are not closely related to the optically accessible morphologies, and this might be why these categorizations did not benefit as profoundly from the multi-measurement strategy. Furthermore, it is indicated that the species-sensitive training drives the ANN to extract more diverse features as the multi-measurement identification of species interpreted into gram-stainability or respiratory metabolism provides higher accuracy than the direct categorization.

## Discussion

We propose a bacterial identification framework that is sensitive to a few individual bacteria, using 3D QPI and ANN. The exceptionally high accuracy under a limited sample quantity is attributable to the remarkable single-cell profiling ability of 3D QPI and the feature-extracting ability of ANN. Results prove that the species-related cellular morphologies captured by 3D QPI are robustly recognized by the trained ANN, remarkably reducing the sample quantity required for identification. Recent studies leveraged ANNs to extract clinically relevant or biologically important information from QPI measurements^26,29–41^. Despite these encouraging results, the capability of 3D QPI and ANN has not been assessed in diagnostic microbiology over a wide variety of species thus far.

We believe that this framework consisting of 3D QPI and ANN can effectively refine the initial antibiotic treatment. The accuracy of species identification using our framework is comparable to that of MALDI-TOF MS^25^, even though the quantity of bacteria involved in the two approaches are single to several cells and over 10^5^ colony-forming units, respectively^42^. In addition, the risk of misidentification based on single tomograms can be strategically suppressed at the cost of specificity. Our framework also shows high single-measurement performance in distinguishing between subgroups of bacteria such as Gram-positive and negative groups. Furthermore, it achieves a nearly perfect identification within the 19 species using only seven tomograms of the bacteria, suggesting that accuracy higher than the single-measurement baseline is viable depending on the situation. Finally, we stress that our framework can be implemented along with the routine microbial identification, including MALDI-TOF MS. That is, the noninvasive property of 3D QPI allows our framework to be added to the existing identification routine without exhausting the initially obtained sample.

Future studies on sample processing will propel our framework towards a more immediate use. In practice, the enrichment of bacteria will be required for 3D QPI measurement when the ratio of bacteria in the given material is extremely small. The concentration of bacteria present in a urine sample is high, and thus the present method can be readily applicable in diagnosing urinary tract infection. On the other hand, bacteria may be scarce in blood samples as well as surrounded by a great number of blood cells. Lysis centrifugation is the common approach to enrich the bacteria from a positive blood culture^43^. However, our sensitive framework can operate before the time-consuming blood culture, if high-throughput sample processing is introduced. A prominent and practical technique is the selective collection of particles utilizing advanced fluidic systems^44–46^, which has successfully demonstrated enrichment of bacteria in laboratory^47,48^.

In addition, validations on a larger diversity of pathogens will expand the scope of application for our method. We expect the proposed framework to be applicable to pathogens causing other classes of infections, such as urinary tract infections and lower respiratory infections, which are partially covered in this study. Moreover, achieving to screen antibiotics-resistant strains will be a crucial step in introducing this framework as a diagnostic routine. It is yet to be assessed whether this framework can distinguish resistant strains, while the need to screen out resistant strains has been highlighted over time^6,49,50^. From a practical point of view, studying and improving ANN’s capability to tolerate the physiological difference is also required to further generalize our method. Although we cultured each species with a fixed protocol and a single type of growth media in this study, each species of bacteria can be cultured or found in various environments. An extreme case would be applying our framework on dead bacterial cells; while our database was collected with live and active bacteria, dead bacterial cells in clinical samples may serve as diagnostic evidence.

Further reducing the cost will encourage extensive studies based on our framework. Even though our framework does not entail an expense as large as MALDI-TOF MS, common hardware implementations of 3D QPI still involve advanced components including a coherent light source, a beam steering device, two microscopic objective lenses, and an imaging sensor with a high space-bandwidth product. Recent studies including Fourier ptychographic tomography^51^ or reference-free intensity-based tomography^52^, have achieved 3D QPI using relatively low-cost and simple optical systems. Despite the differences in the reconstruction process and imaging resolution, these techniques provide sufficient imaging quality for our framework.

The present bacterial species identification framework based on 3D QPI and ANN can also be combined with recently developed techniques of artificial intelligence for image processing, leading to various synergistic studies. For example, an automatic segmentation algorithm^34^ may enable the species identification from densely distributed bacterial samples, such as biofilms^53^ or colonies^54^. Inference of molecular- or chemical-specific information^31–33,55^ can also be exploited for correlative label-free analysis at single-cell or subcellular levels.

Lastly, we expect that the proposed framework will benefit from recent and future advances elucidating the working principle of ANNs. Investigations on ANN architectures have improved the performance of ANNs and expanded the applicability of ANNs over recent years, along with the rapid growth in the hardware capacity. On the other hand, techniques including Bayesian deep learning^56^ have contributed to enhancing the interpretability, as well as offering a guideline for effective optimization. Fostering interpretability will render the proposed method more approachable for the medical industry.

## Materials and methods

### Preparation of bacteria

The bacterial samples were cultured in vitro from frozen glycerol stocks. The frozen stock of each species was stored at −80 °C and thawed at room temperature (25 °C) before use. After thawing, the stock was inoculated into a liquid medium and stabilized for over an hour in a shaking incubator at 35 °C. The stabilized bacteria were seeded in an agar plate containing a suitable medium. The agar plates were incubated at 35 °C for 12−24 h until colony formation was visible. A liquid subculture seeded from the agar plate was incubated at 35 °C for over 8 h in a shaking incubator. The subculture solution was diluted with a liquid medium to a concentration suitable for imaging, then sandwiched between cover glasses. Each species was inoculated in one of the following media: nutrient agar, brain heart infusion agar, tryptic soy agar, and chocolate agar. The glycerol stock or subculture was grown in nutrient broth, brain heart infusion broth, tryptic soy broth, or Giolitti-Cantoni broth.

The specimens were measured alive with no fixation nor any other chemical process; the sample can be immediately measured in the absence of a trained biologist and this is one of the main advantages of this method. A sample slide was prepared by simply sandwiching the solution of bacteria between two cover glasses, after diluting into a concentration suitable for imaging. Before optical measurement, we reduced the turbulent motion in the sample-loaded slides by placing them still on the sample stage for 5–10 min. All of the measurement was carried out within the time window of 8−24 h after inoculating the subculture in order to secure a database of active and live bacteria.

### 3D QPI measurement

We measured each 3D RI tomogram utilizing the 3D QPI as briefly introduced in the Results section. The DMD located on the sample illumination path can alter the illumination angle, by serving as a controllable binary grating^57,58^. Using the DMD, a sinogram of 2D QPI measurements was obtained for each sample by scanning the illumination angle (Fig. 1b). The sinogram covered a total of 49 illumination angles, including a normal angle and 48 oblique angles equally spaced in the azimuthal direction. The 3D RI tomogram was reconstructed from the sinogram under the principle of optical diffraction tomography, which inversely solves the Helmholtz equation^23,59^, then went through an iterative regularization to mitigate the missing cone problem^60^ (Fig. 1c). The detailed procedure for the field retrieval and tomographic reconstruction can be found elsewhere^59,61^.

A continuous-wave laser with a wavelength of 532 nm served as the light source. Two water-immersion objective lenses with 1.2 numerical aperture magnified and de-magnified the light, whereas the polar angle of the oblique illumination was equivalent to a numerical aperture of 0.9. The theoretical resolution of the tomograms was 110 nm in the horizontal direction and 330 nm in the vertical direction, considering the spatial frequency range of the imaging system^62^. The measurement of an entire sinogram required ~0.4 s, which was mainly limited by the camera frame rate.

Each tomogram was cropped into a field of view of 12.8 × 12.8 × 12.8 μm, and sampled at a voxel resolution of 100 × 100 × 200 nm. As a result, each tomogram contained a single bacterium or several bacteria adhering to each other, which considerably depended on the species-related physiology. For instance, specimens of the genus *Streptococcus* were commonly found in chains of multiple bacteria due to their nature.

A manual inspection and curation of tomograms ensured the quality of the database. The quality criteria reflected in this process included the noise level, motion artifact, and location of the specimens. Noisy tomograms, which mostly originated from objects in the oblique illumination path, were removed. Tomograms displaying motion artifacts were also excluded, as turbulent motion faster than the image acquisition rate causes distinctly blurred boundaries. The tomograms were shifted and cropped to place at least one bacterial cell in the central region of the tomogram.

### ANN and optimization

The structure of the ANN in our framework was inspired by a design that outperformed most of the other designs in the benchmark tasks of 2D image analysis^24^. This structure ensures that the feature maps in hidden layers of various depths and scales are utilized for image recognition, by concatenations of the feature maps (Fig. 2a). The elementary units composing our ANN are dense blocks. Each dense block repeats two 3D convolution operations followed by a concatenation (Fig. 2b). The feature maps are re-scaled between two adjacent dense blocks through a transition unit (Fig. 2c). Our ANN included four dense blocks containing 12, 24, 64, and 64 convolution operations, respectively. The number of feature maps after the initial convolution is set to 64, while the number of the feature maps increases by 32 through every convolution operation.

The ANN was optimized to classify the 3D RI tomograms, by minimization of the cross-entropy loss between the ground truth and the prediction. For each species, 40 tomograms were randomly chosen as the blind test dataset and another 40 tomograms were randomly chosen as the validation dataset. The remaining tomograms composed the training dataset, which was directly reflected in the loss minimization process. The loss that occurred in the training dataset was reduced using the stochastic gradient descent algorithm, at a mini-batch size of 48. The step size of the stochastic gradient descent algorithm was scheduled according to the cosine annealing method at an initial step size of 0.001 and a period of 64 epochs^63^. During training, data augmentation took place for each tomogram, once every epoch, to prevent overfitting of the trained model. The augmentation included random processes of a horizontal crop, horizontal rotation, and Gaussian noise. During the blind test, each input tomogram was horizontally cropped around the center to provide an identical dimension. These processes resulted in an input tomogram with a field of view of 9.6 × 9.6 × 12.8 μm to be fed into the ANN. The ANN and the optimization were implemented using PyTorch 1.0.0.

The ANN was trained for ~290 h to obtain the models involved in our results. Two runs of training the ANN from scratch were carried out for ~1000 epochs each. Each training epoch required 504.3 ± 8.3 s in a server equipped with eight graphics processing units (GPUs) of GeForce GTX 1080 Ti and a central processing unit of Xeon E5–2600. The time required to infer a tomogram to a trained ANN model was 28.9 ± 2.9 ms.

Training the ANN with the identical setting can also run on a personal desktop computer, although we utilized an 8-GPU server for training at a higher rate. For instance, a single device of GeForce GTX 1080 Ti is sufficient for training the ANN under our setting, which requires 11,181 MB of graphics memory. When utilizing only a single device of GeForce GTX 1080ti in our server, each training epoch required 516.0 ± 9.6 s. In principle, an ANN of the identical design can be trained with only 1161 MB of graphics memory, by reducing the mini-batch size to 1. However, this minimal setting accompanies 3770.5 ± 67.4 s of duration for a single epoch of training, and altering the mini-batch size may cause the parameters to follow a different path of optimization. For inference using a trained ANN model, 945 MB of graphics memory are sufficient.

The final classifier for the blind test involved the predictions of multiple best-performing ANN models. The models with the highest accuracies for the training and validation datasets were chosen and integrated, to exploit a wider variety of features and prevent model-by-model variance. In search of the optimal strategy for choosing and integrating multiple models, four relevant parameters were explored. These parameters included the number of integrated models, weighting between the accuracies for the training and validation dataset, whether or not to normalize the output, and the method to integrate the predictions by the chosen models. Four options were considered as the method to integrate the predictions: taking the average, taking the exponential average, voting, and taking the maximum projection of the output. The combination of the parameters, which yielded the highest validation accuracy established the algorithm for the blind test.

## Supplementary information

Supplementary Information

## Data Availability

The data that support the findings of this study are available from the corresponding author upon reasonable request.

## References

[1] Hessling M, Feiertag J, Hoenes K (2017). Pathogens provoking most deaths worldwide. Biosci. Biotechnol. Res. Commun..

[2] Torio, C. M. & Moore, B. J. National inpatient hospital costs: the most expensive conditions by payer, 2013. *In: Healthcare Cost and Utilization Project (HCUP) Statistical Briefs [Internet]*. Statistical Brief# 204 (Agency for Healthcare Research and Quality (US), 2016). 27359025

[3] Liu VX (2017). The timing of early antibiotics and hospital mortality in sepsis. Am. J. Resp. Crit. Care Med..

[4] Moehring RW (2013). Delays in appropriate antibiotic therapy for Gram-negative bloodstream infections: a multicenter, community hospital study. PLoS ONE.

[5] García MS (2009). Early antibiotic treatment failure. Int. J. Antimicrobial Agents.

[6] Hutchings MI, Truman AW, Wilkinson B (2019). Antibiotics: past, present and future. Curr. Opin. Microbiol..

[7] Paul M (2010). Systematic review and meta-analysis of the efficacy of appropriate empiric antibiotic therapy for sepsis. Antimicrobial Agents Chemother..

[8] Bizzini A, Greub G (2010). Matrix-assisted laser desorption ionization time-of-flight mass spectrometry, a revolution in clinical microbial identification. Clin. Microbiol. Infect..

[9] Seng P (2009). Ongoing revolution in bacteriology: routine identification of bacteria by matrix-assisted laser desorption ionization time-of-flight mass spectrometry. Clin. Infect. Dis..

[10] Francisco DE, Mah RA, Rabin AC (1973). Acridine orange-epifluorescence technique for counting bacteria in natural waters. Trans. Am. Microsc. Soc.

[11] Müller V (2018). Identification of pathogenic bacteria in complex samples using a smartphone based fluorescence microscope. RSC Adv..

[12] Amann R, Fuchs BM, Behrens S (2001). The identification of microorganisms by fluorescence in situ hybridisation. Curr. Opin. Biotechnol..

[13] Patiño S (2008). Autofluorescence of mycobacteria as a tool for detection of Mycobacterium tuberculosis. J. Clin. Microbiol..

[14] Bhattacharjee A, Datta R, Gratton E, Hochbaum AI (2017). Metabolic fingerprinting of bacteria by fluorescence lifetime imaging microscopy. Sci. Rep..

[15] Park Y, Depeursinge C, Popescu G (2018). Quantitative phase imaging in biomedicine. Nat. Photon..

[16] Mir M (2011). Optical measurement of cycle-dependent cell growth. Proc. Natl Acad. Sci. USA.

[17] Ahn JH (2020). Enhanced succinic acid production by Mannheimia employing optimal malate dehydrogenase. Nat. Commun..

[18] Kemper B (2013). Towards 3D modelling and imaging of infection scenarios at the single cell level using holographic optical tweezers and digital holographic microscopy. J. Biophoton..

[19] Oh J (2020). Three-dimensional label-free observation of individual bacteria upon antibiotic treatment using optical diffraction tomography. Biomed. Opt. Express.

[20] Opota O, Croxatto A, Prod’hom G, Greub G (2015). Blood culture-based diagnosis of bacteraemia: state of the art. Clin. Microbiol. Infect..

[21] Bearman GM, Wenzel RP (2005). Bacteremias: a leading cause of death. Arch. Med. Res..

[22] Lee C-C (2019). Beneficial effects of early empirical administration of appropriate antimicrobials on survival and defervescence in adults with community-onset bacteremia. Crit. Care.

[23] Wolf E (1969). Three-dimensional structure determination of semi-transparent objects from holographic data. Opt. Commun..

[24] Huang, G., Liu, Z., Van Der Maaten, L. & Weinberger, K. Q. Densely Connected Convolutional Networks. *Proc. IEEE Comput. Soc. Conf. Comput. Vis. Pattern Recognit.* 2261–2269 (2017).

[25] Drancourt M (2010). Detection of microorganisms in blood specimens using matrix-assisted laser desorption ionization time-of-flight mass spectrometry: a review. Clin. Microbiol. Infect..

[26] Yoon J (2017). Identification of non-activated lymphocytes using three-dimensional refractive index tomography and machine learning. Sci. Rep..

[27] Hoyer J (2018). Proteomic response of *Streptococcus pneumoniae* to iron limitation. Int. J. Med. Microbiol..

[28] Pathak A (2018). Factor H binding proteins protect division septa on encapsulated Streptococcus pneumoniae against complement C3b deposition and amplification. Nat. Commun..

[29] Jo Y (2018). Quantitative phase imaging and artificial intelligence: a review. IEEE J. Sel. Top. Quantum Electron..

[30] Rivenson Y, Wu Y, Ozcan A (2019). Deep learning in holography and coherent imaging. Light.: Sci. Appl..

[31] Rivenson Y (2019). Virtual histological staining of unlabelled tissue-autofluorescence images via deep learning. Nat. Biomed. Eng..

[32] Kandel ME (2020). Phase imaging with computational specificity (PICS) for measuring dry mass changes in sub-cellular compartments. Nat. Commun..

[33] Jo Y (2021). Label-free multiplexed microtomography of endogenous subcellular dynamics using generalizable deep learning. Nat. Cell Biol..

[34] Choi, J. et al. Label-free three-dimensional analyses of live cells with deep-learning-based segmentation exploiting refractive index distributions. Preprint at *bioRxiv*10.1101/2021.05.23.445351 (2021).

[35] Lee M (2020). Deep-learning-based three-dimensional label-free tracking and analysis of immunological synapses of CAR-T cells. Elife.

[36] Kamilov US (2015). Learning approach to optical tomography. Optica.

[37] Ryu D (2019). Deep learning-based optical field screening for robust optical diffraction tomography. Sci. Rep..

[38] Ryu D (2021). DeepRegularizer: rapid resolution enhancement of tomographic imaging using deep learning. IEEE Trans. Med. Imaging.

[39] Chen CL (2016). Deep learning in label-free cell classification. Sci. Rep..

[40] Ryu, D. et al. Label-free white blood cell classification using refractive index tomography and deep learning. *BME Front.* **2021** (2021). 10.34133/2021/9893804 PMC10521749 37849908

[41] Jo Y (2017). Holographic deep learning for rapid optical screening of anthrax spores. Sci. Adv..

[42] Barreiro JR (2017). Non-culture-based identification of mastitis-causing bacteria by MALDI-TOF mass spectrometry. J. Dairy Sci..

[43] Kirn T, Weinstein M (2013). Update on blood cultures: how to obtain, process, report, and interpret. Clin. Microbiol. Infect..

[44] Lee S (2019). Nanoelectrokinetic bufferchannel-less radial preconcentrator and online extractor by tunable ion depletion layer. Biomicrofluidics.

[45] Kuntaegowdanahalli SS, Bhagat AAS, Kumar G, Papautsky I (2009). Inertial microfluidics for continuous particle separation in spiral microchannels. Lab Chip.

[46] Lei H, Zhang Y, Li B (2012). Particle separation in fluidic flow by optical fiber. Opt. Express.

[47] Jung T, Jung Y, Ahn J, Yang S (2020). Continuous, rapid concentration of foodborne bacteria (*Staphylococcus aureus, Salmonella typhimurium*, and *Listeria monocytogenes*) using magnetophoresis-based microfluidic device. Food Control.

[48] D’Amico L, Ajami N, Adachi J, Gascoyne P, Petrosino J (2017). Isolation and concentration of bacteria from blood using microfluidic membraneless dialysis and dielectrophoresis. Lab Chip.

[49] Shariati A (2020). Global prevalence and distribution of vancomycin resistant, vancomycin intermediate and heterogeneously vancomycin intermediate Staphylococcus aureus clinical isolates: a systematic review and meta-analysis. Sci. Rep..

[50] Chamieh A, El-Hajj G, Zmerli O, Afif C, Azar E (2020). Carbapenem resistant organisms: A 9-year surveillance and trends at Saint George University Medical Center. J. Infect. Public Health.

[51] Horstmeyer R, Chung J, Ou X, Zheng G, Yang C (2016). Diffraction tomography with Fourier ptychography. Optica.

[52] Baek Y, Park Y (2021). Intensity-based holographic imaging via space-domain Kramers–Kronig relations. Nat. Photon..

[53] Berne C, Ellison CK, Ducret A, Brun YV (2018). Bacterial adhesion at the single-cell level. Nat. Rev. Microbiol..

[54] Fenchel T (2002). Microbial behavior in a heterogeneous world. Science.

[55] Nygate YN (2020). Holographic virtual staining of individual biological cells. Proc. Natl Acad. Sci. USA.

[56] Kendall, A. & Gal, Y. What uncertainties do we need in bayesian deep learning for computer vision? *Adv. Neural Inf. Process. Syst.* **30** (2017).

[57] Shin S, Kim K, Yoon J, Park Y (2015). Active illumination using a digital micromirror device for quantitative phase imaging. Opt. Lett..

[58] Lee K, Kim K, Kim G, Shin S, Park Y (2017). Time-multiplexed structured illumination using a DMD for optical diffraction tomography. Opt. Lett..

[59] Kim K (2013). High-resolution three-dimensional imaging of red blood cells parasitized by *Plasmodium falciparum* and in situ hemozoin crystals using optical diffraction tomography. J. Biomed. Opt..

[60] Lim J (2015). Comparative study of iterative reconstruction algorithms for missing cone problems in optical diffraction tomography. Opt. Express.

[61] Debnath SK, Park Y (2011). Real-time quantitative phase imaging with a spatial phase-shifting algorithm. Opt. Lett..

[62] Park C, Shin S, Park Y (2018). Generalized quantification of three-dimensional resolution in optical diffraction tomography using the projection of maximal spatial bandwidths. J. Opt. Soc. Am. A.

[63] Loshchilov, I. & Hutter, F. Sgdr: Stochastic gradient descent with warm restarts. Preprint at https://arxiv.org/abs/1608.03983 (2016).

