# Supplementary material for: Rapid species identification of pathogenic bacteria from a minute quantity exploiting three-dimensional quantitative phase imaging and artificial neural network
Source: Light Sci Appl. 2022 Jun 23;11:190. doi: 10.1038/s41377-022-00881-x (PMC9226356; doi:10.1038/s41377-022-00881-x)
Supplement: Supplementary file 1 — Supplementary Information [file 41377_2022_881_MOESM1_ESM.docx]

**Supplementary Information for**

Rapid species identification of pathogenic bacteria from a minute quantity exploiting three-dimensional quantitative phase imaging and artificial neural network

Geon Kim^1,2^, Daewoong Ahn^3^, Minhee Kang^4^, Jinho Park^1,2^, DongHun Ryu^1,2^, YoungJu Jo^1,2,3,a^, Jinyeop Song^1,2,b^, Jea Sung Ryu^5^, Gunho Choi^3^, Hyun Jung Chung^5,6^, Kyuseok Kim^7^, Doo Ryeon Chung^8^, In Young Yoo^9^, Hee Jae Huh^10^, Hyun-seok Min^3^, Nam Yong Lee^10,*^, and YongKeun Park^1,2,3,*^

# ^1^Department of Physics, Korea Advanced Institute of Science and Technology, Daejeon 34141, Republic of Korea.

# ^2^KAIST Institute for Health Science and Technology, KAIST, Daejeon 34141, Republic of Korea

# ^3^Tomocube Inc., Daejeon 34109, Republic of Korea.

# ^4^Smart Healthcare & Device Research Center, Samsung Medical Center, Sungkyunkwan University School of Medicine, Seoul 06351, Republic of Korea.

# ^5^Graduate School of Nanoscience and Technology Korea Advanced Institute of Science and Technology, Daejeon 34141, Republic of Korea.

# ^6^Department of Biological Science, Korea Advanced Institute of Science and Technology, Daejeon 34141, Republic of Korea.

# ^7^Department of Emergency Medicine, Bundang CHA Hospital, Gyeonggi-Do 13496, Korea.

# ^8^Division of Infectious Diseases, Department of Internal Medicine, Samsung Medical Center, Sungkyunkwan University School of Medicine, Seoul 06351, Republic of Korea.

# ^9^Department of Laboratory Medicine, Seoul St. Mary's Hospital, College of Medicine, The Catholic University of Korea, Seoul 06591, Republic of Korea.

# ^10^Department of Laboratory Medicine and Genetics, Samsung Medical Center, Sungkyunkwan University School of Medicine, Seoul 06351, Republic of Korea.

# ^a^Present address: Department of Applied Physics, Stanford University, Stanford, CA 94305, United States

# ^b^Present address: Department of Physics, Massachusetts Institute of Technology, Cambridge, MA 02139, United States

^*^Corresponding authors: micro.lee@samsung.com and yk.park@kaist.ac.kr

**1. Quantitative biophysical properties of bacteria**

**
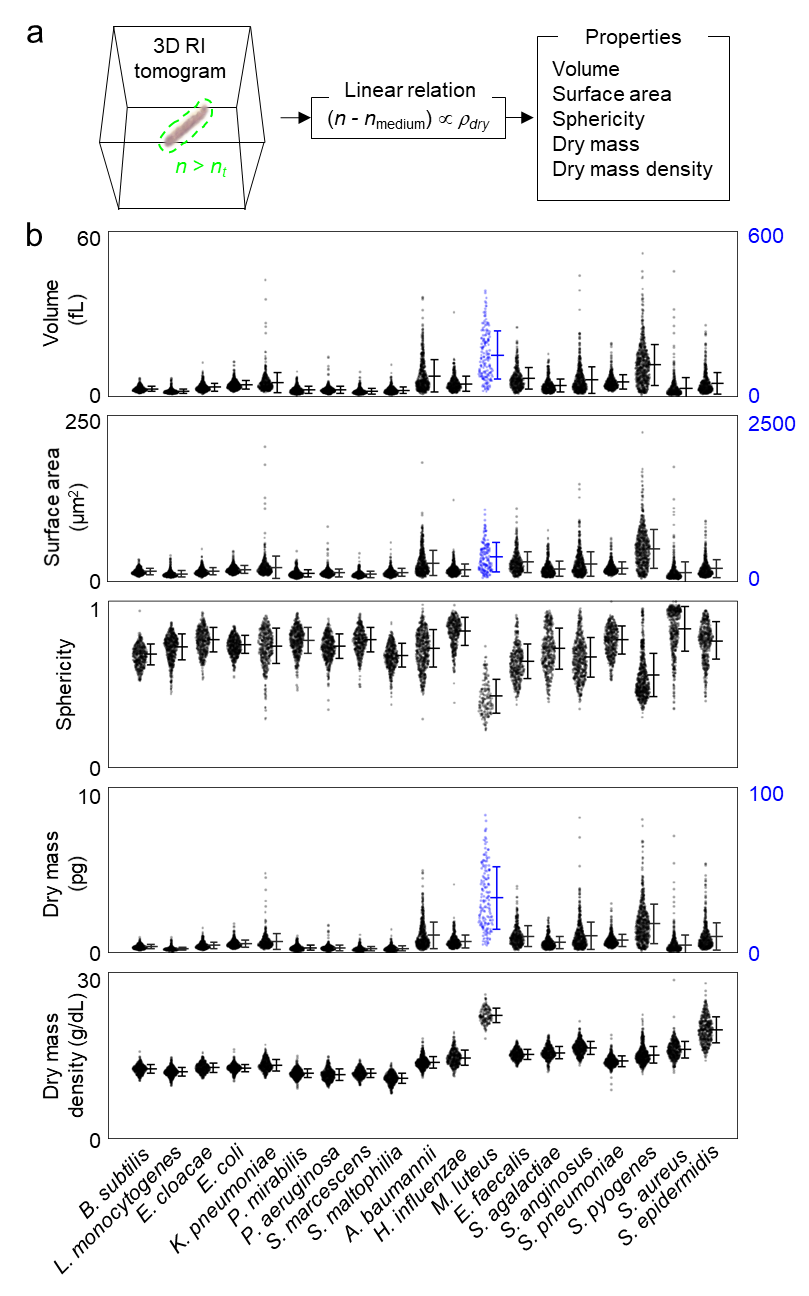
**

**Fig. S1. Biophysical properties of bacteria obtained from individual 3D RI tomograms.** **a** Obtaining the biophysical properties from a 3D RI tomogram. **b** The scatter plot of biophysical properties of each species, obtained with *n_t_* = 1.35. Due to significant differences in the range of the properties, the volume, surface area, and dry mass of *M. luteus* are plotted in different scales, indicated with blue color.

Quantitative biophysical properties of bacteria can be obtained from 3D RI tomograms, since RI is a quantitative contrast related to the material content. The bacteria in a 3D RI tomogram can be segmented from the background based on an RI threshold, thus the volume, surface area, and sphericity can be calculated from the segmentation map. Furthermore, the total dry mass and the average dry mass density can be calculated from the RI distribution inside the bacteria, based on the linear relation between the RI and solvent concentration in aqueous solutions like cells^1, 2^ (Fig. S1a).

The biophysical properties that were obtained based on the RI threshold of *n_t_* = 1.35 visualized the statistic distribution of each species (Fig. S1b). While characteristic distributions of biophysical properties were visible for each species, the properties were highly overlapping between most of the species with an exception of *M. luteus,* which was displayed significantly large dimension and dry content.

**2. Suitability of 3D QPI for species identification of bacteria**

**
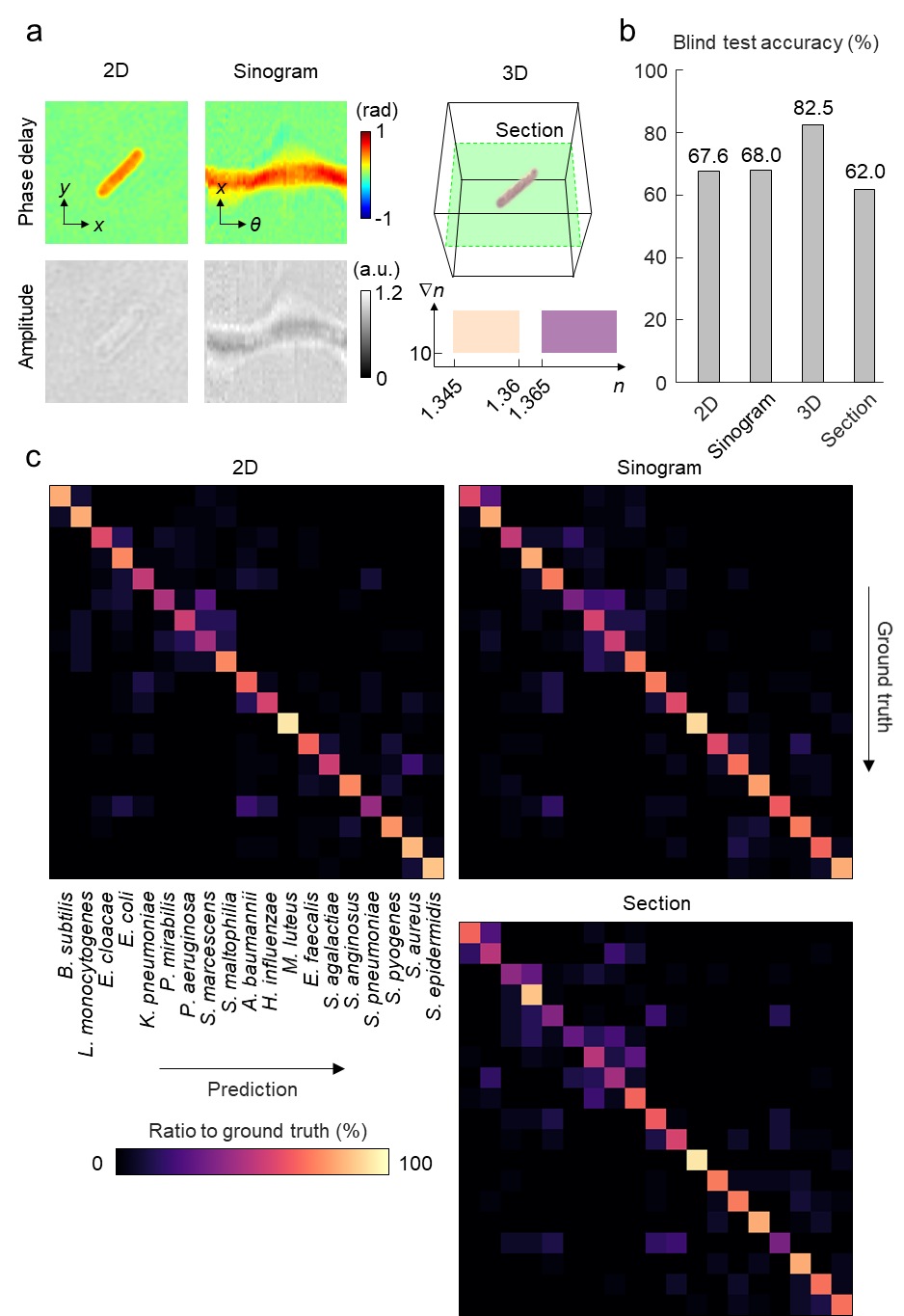
**

**Fig. S2. Comparison between 2D and 3D QPI in ANN-based species identification using a single image. a** Multiple types of QPI data are compared in the task of image-based species identification: 2D QPI, sinogram, 3D QPI, and the focal section of 3D QPI. **b** The overall accuracy resulting from four approaches in **a**. **c** The confusion matrices of species identification for all types of data, except for 3D QPI.

The benefit of 3D QPI in the identification of bacteria at a single-CFU level was verified in a comparative experiment. The proposed framework based on 3D QPI was compared with two variant approaches that also include ANNs but are based on 2D QPI. In one approach, the ANN identifies the species from the 2D QPI measurement, while in the other the ANN identifies the species from the sinogram of 2D QPI measurements in multiple illumination angles (Fig. S2a). Furthermore, a similar comparison was carried out using the focal slice of the 3D RI tomogram to explore a computationally simpler approach. Here, the vertical section of a 3D RI tomogram with the highest average RI was selected as the focal section to provide the largest image contrast.

The approaches based on 2D QPI, the sinogram and the focal section of the tomogram achieved 67.6%, 68.0%, and 62.0% blind-test accuracy respectively (Fig. S2b), after training the ANN and integrating high-performing models. The significant difference in the accuracy suggests that 3D QPI offers species-related features to ANN in a more ostensive manner compared to 2D QPI or sinogram.

When using 2D QPI data, the significant difference in identification performance included 2.18 times higher among gram-negative bacilli and 4 times higher among gram-negative coccobacilli, compared to when using 3D QPI data. The most frequent error was the misidentification of P. mirabilis into S. marcescens, which was 3.67-fold more frequent compared to the 3D QPI case.

When using the sinogram data, the confusion within each of the four main taxonomical groups all increased approximately 2 times. The most frequent error was the misidentification of B. subtilis into L. monocytogenes, which was 3.67-fold more frequent compared to the 3D QPI case.

When using the focal section of the tomogram, the significantly higher error compared to the using the entire tomogram was the confusion among gram-negative coccobacilli which increased 3 times. The most frequent error was the misidentification of E. cloacae into E. coli (30.0%), which was 6-fold more frequent than the approach using the entire 3D RI tomograms.

**3. Suitability of our ANN for classification of 3D RI tomograms**

**
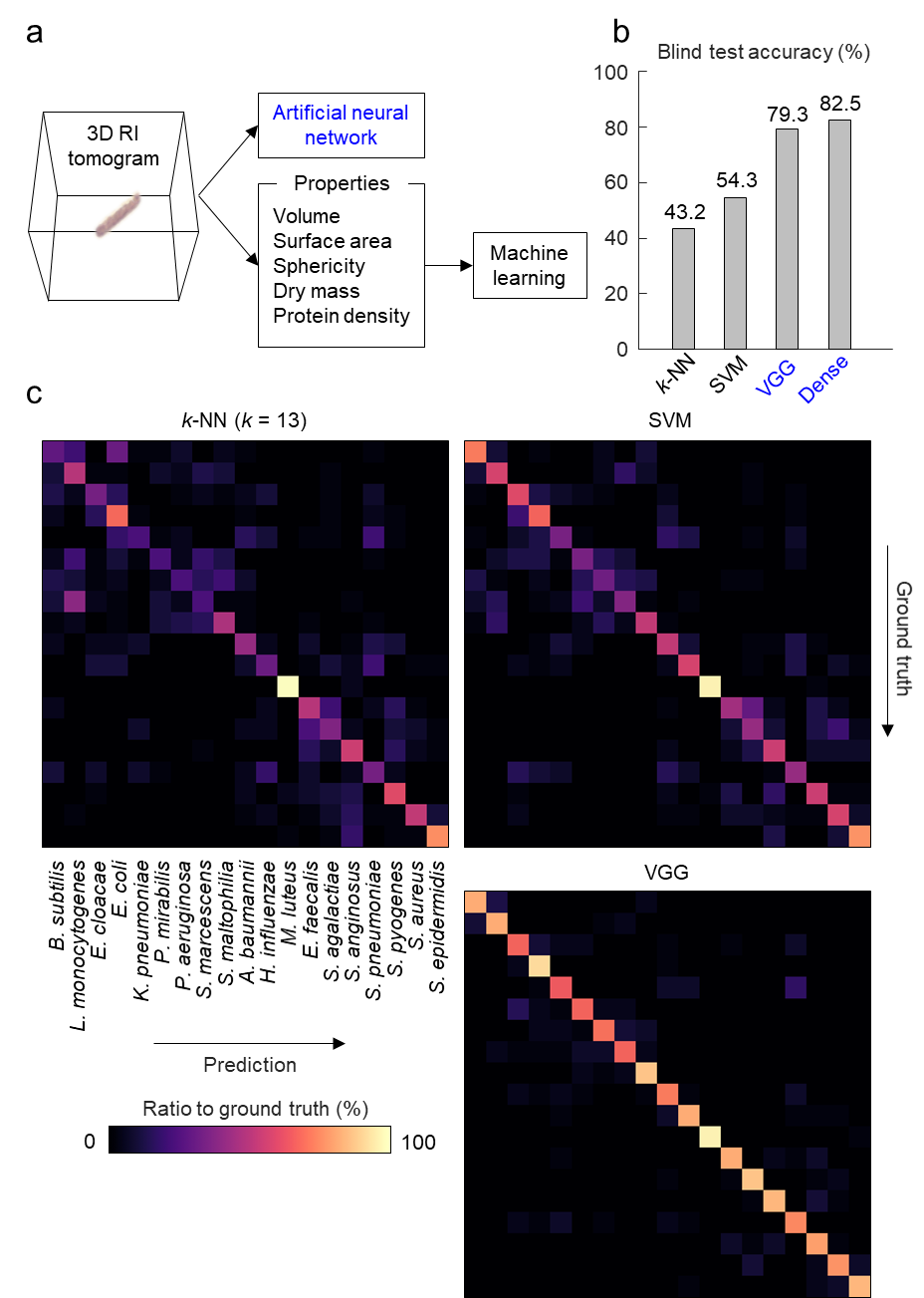
**

**Fig. S3. Comparison between the implemented ANN and conventional machine learning in species identification from 3D QPI data.** **a** The schematic of ANN-based end-to-end learning and machine learning with hand-crafted features. **b** The identification accuracy of four species identification approaches: *k*-NN with hand-crafted features, SVM with hand-crafted features, a simpler ANN, and our ANN with the 3D RI tomogram. The case of *k* =13, which displayed the highest accuracy, is shown for *k*-NN. The simpler ANN is a VGGNet structure with 11 layers, while our ANN is based on the DenseNet structure with 169 layers. The SVM approach involves 171 binary SVM classifiers of linear kernels. **c** The confusion matrices of species identification based on the three approaches other than the implemented ANN.

The performance of our ANN in identifying the species of bacteria from the 3D RI tomogram was compared to that of conventional machine learning approaches, as well as another ANN structure which is simpler and prevalently used. Two of the most commonly implemented machine learning techniques, *k*-nearest neighbor algorithm (*k*-NN) and support vector machine (SVM), were selected for the comparison. For the hand-crafted features, biophysical properties retrieved using multiple RI threshold values were concatenated, where the threshold values were *n_t_* = 1.34, 1.342, 1.344, ..., 1.378^3^. Each feature element was standardized to have 0 mean and 1 standard deviation (Fig. S3a). As the other ANN structure, we chose a VGGNet structure with 8 convolutional layers and 2 fully-connected layers^4^. The VGGNet structure is relatively simple and prevalently used, therefore was considered a suitable structure for comparison. The 8 convolutional layers were of 3×3×3 kernels, with output feature map sizes of 16, 32, 64, 64, 128, 128, 128, and 128. Max pooling layers with 2×2×2 windows and a stride of 2 were placed after the first, second, fourth, sixth, and eighth convolutional layers. The output feature size of the two fully-connected layers were 1024 and 19, where the latter indicated the 19 species.

In general, the confusion of machine learning between the bacilli accounted for a significant portion of the error similarly to the ANN (Fig. S3b,c). Yet the confusion between the cocci was comparably high, despite that ANN did not suffer as significant error between cocci.

For *k*-NN, the number of nearest neighbors was scanned from *k* = 1 to *k* = 40, resulting in the highest accuracy (43.2%) at *k* = 13 and the lowest accuracy (34.3%) at *k* = 1, 2. A major tendency compared to ANN was the 3.47 times higher error rate between gram-positive cocci. The most profound error was the misidentification of *S. marcescens* into *L. monocytogenes* (37.5%), which was 15-fold more frequent compared to the identical misidentification error using ANN. The second most common misidentification was *B. subtilis* into *E. coli* (30.0%), which the ANN did not suffer.

For SVM, a multi-class classifier was established by integrating the results of 171 binary classifiers, each of which is trained to distinguish between two species. Two integration strategies of simple voting and score-weighted voting, as well as two types of kernels, linear and Gaussian, were employed and compared. The highest accuracy of SVM (54.3%) was achieved using simple voting and linear kernel, whereas the lowest accuracy (17.5%) resulted from score-weighted voting and Gaussian kernel.

For SVM, which we have additionally implemented during the revision, a major tendency compared to ANN was the 3.30 times higher error rate between gram-positive cocci and the 3.00 times higher error rate between gram-negative coccobacilli. The most profound error was the misidentification of *E. faecalis* into *S. agalactiae*, which the ANN did not suffer. Other frequent errors included the misidentifications of *S. agalactiae* into *S. aureus*, *S. marcescens* into *P. mirabilis*, and *E. coli* into *E. cloacae* (25.0%) which were 2-, 8- and 4-fold more frequent compared to those of ANN respectively.

Using the VGGNet to classify the 3D RI tomogram achieved 79.3% accuracy, which was moderately lower than using our ANN based on a DenseNet with 169 layers. Overall, the errors similar to those resulting from our ANN occurred to the VGGNet classification, except for an increase in several specific cases of confusion. The most frequent confusion was the misidentification of *K. pneumoniae* into *S. pneumoniae* (17.5%) which was also a significant confusion for our ANN. Other significant cases of confusion included the misidentification of *P. mirabilis* into *E. cloacae* (15.0%) and the misidentification of *B. subtilis* into *L. monocytogenes* (12.5%).

**4. Effectiveness of hand-crafted features**

**
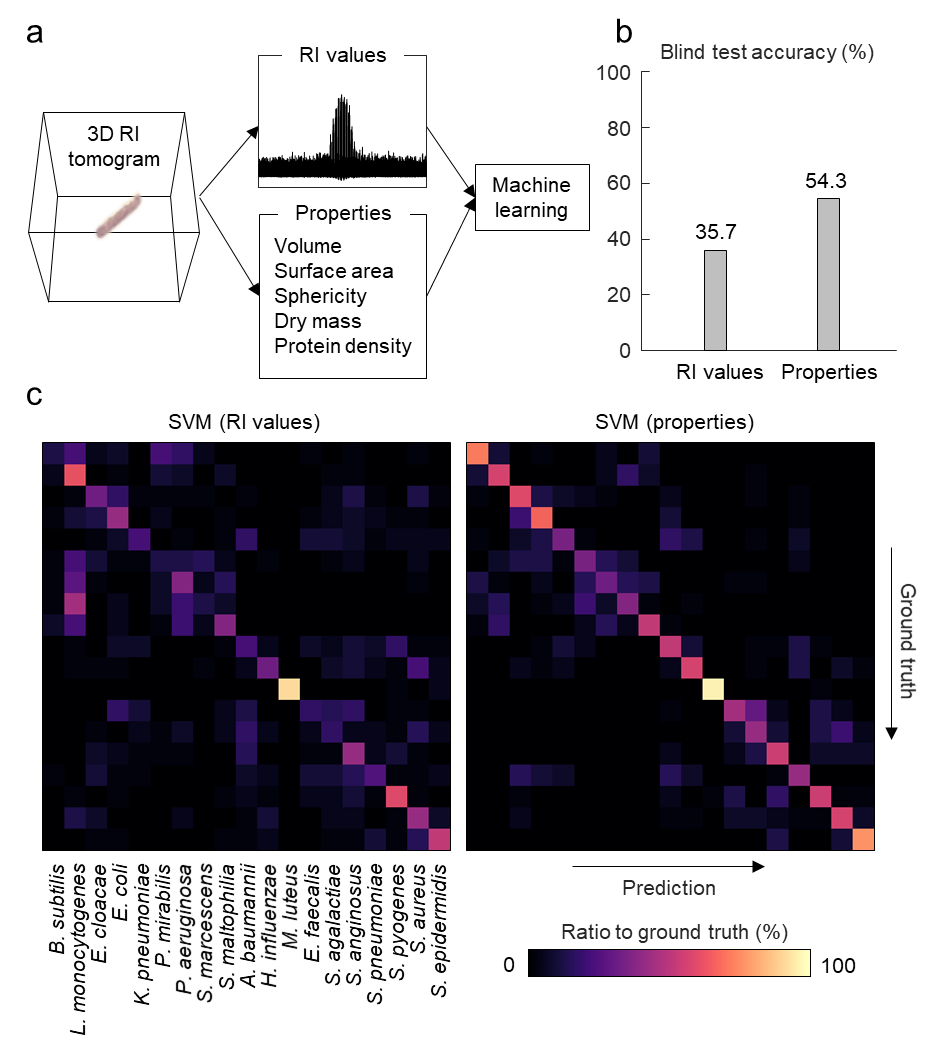
**

**Fig. S4. Comparison between the entire 3D RI tomogram and the hand-crafted features used in SVM-based species identification. a** The schematic of machine learning with all the RI values in a tomogram (upper) and hand-crafted features (lower). **b** The identification accuracy of the two approaches. Both approaches utilize 171 binary SVM classifiers of linear kernels. **c** The confusion matrices of species identification based on the two approaches.

The effectiveness of the hand-crafted features, that were compared along the ANN analysis in section 3, was verified by performing machine learning classification without the feature extraction. To be specific, all the RI values of 589,824 voxels in each 3D RI tomogram were reshaped into a linear vector and utilized as the features for SVM classification (Fig. S4a). In the identical setting of classifier voting and a linear kernel, the classification based on all the RI values was 35.7% accurate, which was significantly lower than the accuracy of 54.3% achieved using the hand-crafted features (Fig. S4b). This difference in the identification performance implies that

The classification without hand-crafted feature extraction was less accurate for most of the 19 species with small exceptions (Fig. S4c). 16 out of the 19 species were identified with reduced sensitivity when classifying with all the RI values. The sensitivity of identifying *B. subtilis* displayed the largest reduction from 72.5% to 12.5%. *L. monocytogenes*, *P. aeruginosa*, and *S. pyogenes* were the three remaining species, each of which displayed an increase of 5.0% in sensitivity. The largest increases in the identification error included the misidentification of *S. marcescens* into *L. monocytogenes* (from 15.0% to 45.0%) and *P. aeruginosa* into *L. monocytogenes* (from 2.5% to 27.5%). However, decreases in the error were also observed, including the misidentification of *L. monocytogenes* into *S. marcescens* (0% to 17.5%) and *E. faecalis* into *S. agalactiae* (15.0% to 30.0%).

**5. Contrast in ANN output**


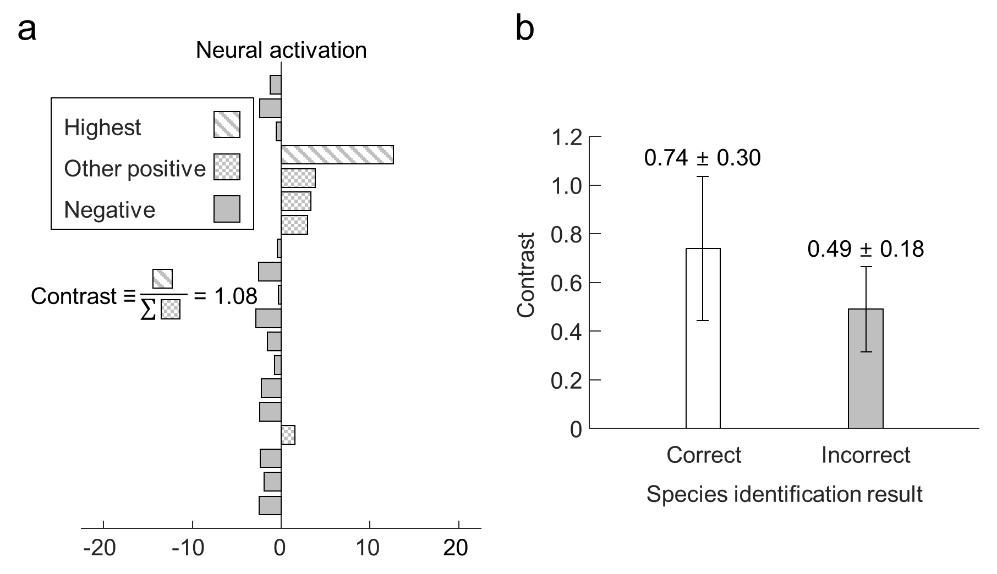


**Fig. S5. Contrast in the ANN output for correctly and incorrectly identified tomograms.** The contrast of the ANN output is defined as the highest output value divided by the sum of other positive output values, to represent the exclusiveness of the identification. **a** an example of assessing the contrast in the ANN output. **b** Statistical comparison of the output contrast between the correctly identified test data and the misidentified test data.

Our trained ANN robustly extracts species-related features from 3D RI tomograms, even in misidentified cases. We were able to discern this phenomenon from the contrast in the ANN outputs; the ANN predicted with more certainty in the correctly identified cases. The contrast in the ANN output, defined as the highest activation value divided by the sum of all other positive activation values (Fig. S5a) was significantly higher in the correctly identified cases than the misidentified cases (Fig. S5b). Because a high output value is present in the correct species as well as the predicted species, the ANN outputs of misidentified cases displayed relatively low contrasts.

**6. Sensitivity and specificity in identifying each species**

**
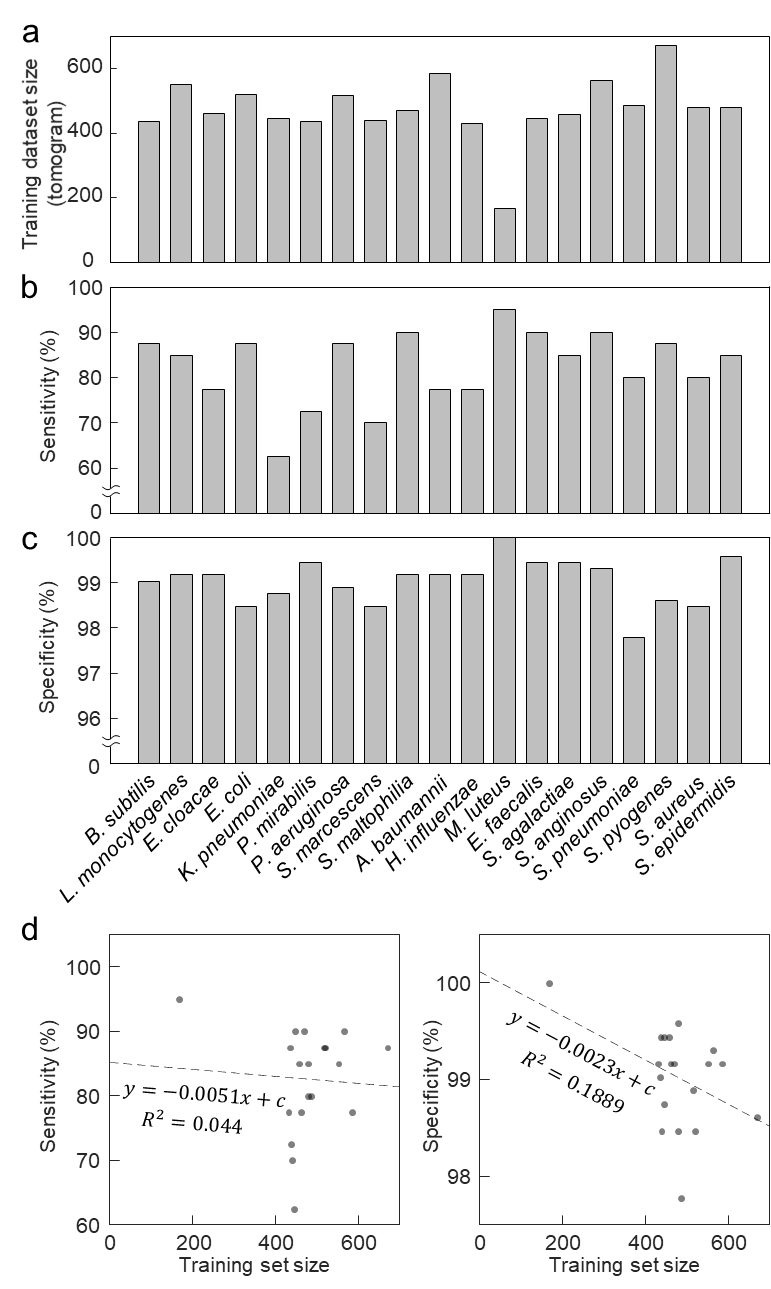
**

**Fig. S6. The number of training dataset and identification performance of each species.** **a** The number of training data for each species of our study. **b** The sensitivity of correctly identifying each species using our framework. **c** The specificity of not correctly excluding each species using our framework. **d** The correlation of the sensitivity and specificity to the number of the training set size.

In order to assess the species-wise distribution of the accuracy, we carried out an additional analysis of the identification result, as well as their relations with the distribution of the training dataset size (Fig. S6). The correlation between the performance in identifying each species and the size of its training dataset was not significant. Both the sensitivity and specificity displayed marginally negative correlations with the size of the training dataset, while the low *R*^2^ values suggested weak correlations. This implies that the variation of identification performance among different species is more dependent to the species-related distinction in the 3D RI tomograms, rather than the size of the training dataset.

**References**

1. Barer R. Determination of dry mass, thickness, solid and water concentration in living cells. *Nature* 1953, **172**(4389)**:** 1097-1098.

2. Barer R. Interference microscopy and mass determination. *Nature* 1952, **169**(4296)**:** 366-367.

3. Yoon J, Jo Y, Kim M-h, Kim K, Lee S, Kang S-J*, et al.* Identification of non-activated lymphocytes using three-dimensional refractive index tomography and machine learning. *Scientific reports* 2017, **7**(1)**:** 1-10.

4. Simonyan K, and Zisserman A. Very deep convolutional networks for large-scale image recognition. *arXiv preprint* 2014, arXiv:1409.1556.
